# ε Subunit of *Bacillus subtilis* F_1_-ATPase Relieves MgADP Inhibition

**DOI:** 10.1371/journal.pone.0073888

**Published:** 2013-08-13

**Authors:** Junya Mizumoto, Yuka Kikuchi, Yo-Hei Nakanishi, Naoto Mouri, Anrong Cai, Tokushiro Ohta, Takamitsu Haruyama, Yasuyuki Kato-Yamada

**Affiliations:** 1 Department of Life Science, Rikkyo University, Tokyo, Japan; 2 Research Center for Life Science, Rikkyo University, Tokyo, Japan; University of Saskatchewan, Canada

## Abstract

MgADP inhibition, which is considered as a part of the regulatory system of ATP synthase, is a well-known process common to all F_1_-ATPases, a soluble component of ATP synthase. The entrapment of inhibitory MgADP at catalytic sites terminates catalysis. Regulation by the ε subunit is a common mechanism among F_1_-ATPases from bacteria and plants. The relationship between these two forms of regulatory mechanisms is obscure because it is difficult to distinguish which is active at a particular moment. Here, using F_1_-ATPase from *Bacillus subtilis* (BF_1_), which is strongly affected by MgADP inhibition, we can distinguish MgADP inhibition from regulation by the ε subunit. The ε subunit did not inhibit but activated BF_1_. We conclude that the ε subunit relieves BF_1_ from MgADP inhibition.

## Introduction

FoF_1_-ATPase/synthase (FoF_1_) catalyzes ATP synthesis from ADP and inorganic phosphate coupled with the flow of H^+^ driven by the electrochemical gradient of H^+^ across cellular membranes. FoF_1_ consists of a water-soluble ATP-driven F_1_ motor (F_1_-ATPase) connected to a membrane-embedded H^+^-driven Fo motor to couple ATP synthesis/hydrolysis and H^+^ flow via a unique rotary mechanism [1–4]. F_1_-ATPase comprises α_3_, β_3_, γ, δ and ε subunits and its hydrolysis of one ATP molecule drives a discrete 120° rotation of the γε subunits relative to the others [5,6]. In FoF_1_, rotation of the rotor subunits of F_1_ (γ and ε) is transferred to the c subunit-ring of Fo to couple ATP synthesis/hydrolysis and flow of H^+^.

The smallest subunit, ε, is an endogenous inhibitor of the ATPase activity of bacterial and chloroplast F_1_-ATPases and is believed to contribute to the regulation ATP synthase [7–10]. The mechanism of inhibition by the ε subunit (ε inhibition) varies among species. For example, when F_1_-ATPase is separated from Fo, the ε subunit works as a dissociative inhibitor in *Escherichia coli* (EF_1_) and plant chloroplasts (CF_1_). The ε subunit inhibits ATPase activity, and the enzyme is reactivated when it dissociates from F_1_-ATPase, and the addition of excess ε subunits restores inhibition. In contrast, the ε subunit of F_1_-ATPase from thermophilic *Bacillus* PS3 (TF_1_) does not dissociate from the TF_1_ complex, and the addition of excess ε subunits does not significantly inhibit activity [10]. Rather, the ε subunit controls the activation state of the enzyme by changing its conformation. Because the dissociation of the ε subunit may not occur within the ATP synthase holo-complex, the ε subunit of EF_1_ or CF_1_ may also work as a regulator in intact ATP synthase. When in the extended conformation, the C-terminal domain of the ε subunit elongates into the cavity of the α_3_β_3_ ring and inhibits ATPase activity [11–16]. Upon activation, the C-terminal α helices of the ε subunit are expelled from the α_3_β_3_ ring and the ε subunit takes a folded-state conformation in which the C-terminal α helices are folded into a helix-turn-helix conformation, and ATPase activity is not inhibited [17]. We recently demonstrated that, in the case of TFoF_1_, the coupling between ATPase activity and flow of H^+^ is altered when the ε subunit does not bind ATP [18].

F_1_-ATPase is most commonly regulated by MgADP inhibition [19–21], which affects all known ATP synthases, and it is caused by the entrapment of MgADP at the catalytic site(s). The recovery from MgADP inhibition is accelerated when ATP binds to non-catalytic sites [22–26]. MgADP inhibition can be observed as pauses of the rotation of the γ subunit [27]. The pause angle of the γ subunit during MgADP inhibition is the same as that of the catalytic dwell (80° from the ATP-binding dwell), which is also the same as that during ε inhibition [28–30]. From this and other results, some investigators have proposed that ε inhibition is caused by the stabilization of MgADP inhibition [28,29,31]. Conversely, ε inhibition is prominent even in the presence of the detergent, lauryl dimethyl amine oxide (LDAO) (see supplemental figures of ref[14].), which is known to reduce MgADP inhibition [32]. Further, MgADP inhibition occurs even in the absence of the ε subunit. We demonstrated that the ε subunit greatly reduces the affinity of catalytic sites for MgATP and MgADP [10,33], which counteracts MgADP inhibition rather than stabilizing it. We have shown that the ε subunit relieved MgADP inhibition of a mutant TF_1_ unable to bind nucleotides to non-catalytic sites although at low levels [30]. Sekiya et al. reported that the ε subunit does not significantly influence MgADP inhibition of *E. coli* F_1_-ATPase [34]. Konno et al. proposed the existence of different origins of MgADP inhibition and ε inhibition in cyanobacterial F_1_-ATPase [35]. This discrepancy may be explained by concurrent and indistinguishable MgADP inhibition and ε inhibition.

Although the FoF_1_-ATP synthase from *Bacillus subtilis* has been studied for decades [36–38],, to the best of our knowledge, no detailed kinetic analysis of the purified enzyme has been reported, particularly regarding ε inhibition or MgADP inhibition. To address this question, in the present study, we purified F_1_-ATPase from *B. subtilis* (BF_1_) and carried out detailed analyses of the relationship between MgADP inhibition and the function of the ε subunit. Because the activity of BF_1_ is strongly affected by MgADP inhibition, we were able to examine the effect of the ε subunit on MgADP inhibition in detail. The results clearly indicate that regulation by the ε subunit is not only distinct from MgADP inhibition but their effects counteract each other.

## Materials and Methods

### Construction of a Plasmid to Express the α_3_β_3_γ Complex of BF_1_

KOD-Plus DNA DNA polymerase (Toyobo) was used for PCR reactions. The region containing the genes encoding the α, γ, and β subunits of BF_1_ was amplified by genomic PCR by using two primers as follows: 5′-CCGAATTCATATGAGCATCAAAGCTGAAGAGATTAGCACGC-3′ contains *Eco*RI and *Nde*I sites. The initiation codon of the α subunit (GTG) was replaced with ATG; 5′-GGCTCGAGCTGCAGTTAAACTTCTACACCCATTTCTTTTGCTTTC-3′ contains *Pst*I and *Xho*I sites and the termination codon for the β subunit. *B. subtilis* genomic DNA was used as template. The PCR product was cloned into the *Eco*RV site of the pZero2.1 vector (Invitrogen) to produce pZero-BF1. The initiation codon of the γ subunit was converted from TTG to ATG and the SD sequence of the γ subunit was converted from AAGG to AAGGAGG, as reported for the expression system of TF_1_ [39] using overlap-extension PCR [40,41] with the four primers as follows: The mutagenic primers, 5′-AGAGAAAAGGAGGTGAAATCCATGGCCTCATTACG-3′ and 5′-AATGAGGCCATGGATTTCACCTCCTTTTCTCTTC-3′ contain an *Nco*I site in addition to the modifications described above; flanking primers were 5′-GCTGTCCTTGCTTCTTCGCCGTCCGC-3′ and 5′-TCTTGTGTGATGGCTGCTTGGCGAG-3′. The resulting 1.6-kbp fragment, containing segments of the genes encoding the γ and α subunits, was cloned into the *Eco*RV site of pZero2.1. A 1-kbp *Bgl*II fragment containing the initiation codon for the γ subunit was transferred to the cognate site of pZero-BF1 in the correct orientation to generate pZero-BF1ATG. The full-length genes encoding the α, γ, and β subunits were excised from pZero-BF1ATG with *Nde*I and *Xho*I and cloned into the respective sites of the pET16b expression vector (Novagen), generating pET16b-BF1 in which a His_10_-tag was introduced at the N-terminus of the α subunit. However, we were unable to express or purify the α_3_β_3_γ complex of BF_1_ from this construct as most of the α subunits were expressed as monomers. To introduce the His_6_-tag at the N-terminus of the β subunit, overlap-extension PCR was carried out using the four primers as follows: The mutagenic primers containing the His_6_-tag were 5′-CGATGCATCATCATCATCATCACATGAAGAAAGGACGCGTTAGCCAGG-3′ and 5′-CTTCATGTGATGATGATGATGATGCATCGCTATCCCTCCTGACAAAATC-3′. The flanking primers were 5′-CAGTTCGGTTTTACGGAGTGCTTATC-3′ and 5′-GCGCCGGGTCAGTGTAGTCATCG-3′. The resulting 1.6-kbp fragment, which contained the region around the initiation codon for the β subunit, was cloned into the *Eco*RV site of pZero2.1 to generate pZero2.1-βhis. To remove the His_10_-tag at the N-terminus of the α subunit, the *Nde*I/*Xho*I-digested fragment of pET16b-BF1, which contains the genes for the α, γ, and β subunits, was transferred to the respective sites of pET21a (Novagen) to produce pET21-BF1nohis. Then, to introduce the His_6_-tag into the β subunit in pET21-BF1nohis, pZero2.1-βhis was digested with *Bse*RI/*Bss*SI. A 0.8-kbp fragment, which contained the N-terminus of the β subunit was isolated and ligated to a 7.2-kbp fragment of pET21-BF1nohis-digested with *Bse*RI/*Dra*III, and a 1.3-kbp fragment, which contained most of the β subunit gene of pET21-BF1nohis, which was digested with *Bss*SI/*Dra*III, to obtain pET21-BF1. The final product, pET21-BF1, contained the following modifications of the original genes as follows: the His_6_-tag was introduced at the N-terminus of the β subunit, the initiation codon of the γ subunit was replaced with ATG, the SD sequence of the γ subunit was modified, an *Nde*I site was introduced at the 5′-terminus of the α subunit gene, and an *Nco*I site was introduced at the 5′-terminus of the γ subunit gene.

### Construction of plasmids to express mutant proteins

The plasmid expressing the mutant (γ^S3C^) α_3_β_3_γ complex of BF_1_ was constructed as follows. The sequence of the genes encoding the entire γ subunit and part of the β subunit were amplified by PCR from pET21-BF1 with the primers as follows. The mutagenic primer was 5′-AAATCCATGGCCTGTTTACGCGATATTAAG-3′, which contains a γSer3 to Cys substitution, and the other primer was 5′-ATGTAAGGAGCAAGCAAATCAACAAC-3′. The resulting 1.3-kbp fragment was introduced into the *Eco*RV site of pZero2.1 to produce pZero-γS3C. Then, pZero-γS3C was digested with *Nco*I/*Sal*I, and a 1-kbp fragment containing the γ^S3C^ region was recovered and ligated to a 7.4-kbp fragment of pET21-BF1 digested with *Mun*I/*Sal*I, and a 0.9-kbp fragment of *Mun*I/*Nco*I-digested pET21-BF1, which contained a segment of the gene encoding the α subunit, was ligated to obtain pET21-BF1 (γS3C). The plasmid expressing a mutant BF_1_ ε subunit (133C, where a Cys was introduced at the C-terminus) was constructed using the following primers for PCR as follows: 5′-GCCGGCGAAGCTTAACATTTCCCTGCTAC-3′, which contains 133C at the C-terminus of the BF_1_ ε subunit and a *Hind*III site, and 5′-GAAATTAATACGACTCACTATAGG-3′, which corresponds to the upstream sequence of the gene encoding the BF_1_ ε subunit (T7 promoter). The expression plasmid for WT BF_1_ ε [42] was used as the template. The resulting DNA fragment was cloned into the *Eco*RV site of pZero2.1; the resulting plasmid was digested with *Nde*I/*Hind*III, and the DNA fragment was transferred to the respective cognate sites of the pET21b expression vector to produce pET21-BF1ε(133C).

### Protein purification

WT or mutant (γ^S3C^) α_3_β_3_γ complexes of BF_1_ were prepared as follows: *E. coli* BL21(DE3) was transformed with pET21-BF_1_ and grown in 1-L LB medium containing 100 mg/L ampicillin and 10 µM IPTG at 25° C for 24–36 h with vigorous shaking at 250 rpm in a 3-L baffled flask. Typically, approximately 6 g wet cells was produced. Cells were suspended in buffer A (20 mM Tris-H_2_SO_4_ (pH 7.5), 300 mM K_2_SO_4_, and 30 mM imidazole) to 0.1–0.2 g cells/ml and disrupted using a French Press. The rest of the procedures was carried out at 25° C. Cell debris was removed by centrifugation at 2,000 × *g* for 15 min at 25° C. The supernatant was diluted with the same volume of buffer A and applied to a 5 ml HisTrapFF crude column (GE Healthcare Life Sciences) equilibrated with buffer A at a flow rate at 2 ml/min. The column was washed with buffer A until the absorbance at 280 nm plateaued. The adsorbed proteins were eluted with buffer B (buffer A containing 500 mM imidazole) and collected. Fractions were purified using a gel-filtration column (Superdex 200 10/300 GL; GE Healthcare Life Sciences) equilibrated with buffer C (50 mM Tris-H_2_SO_4_ (pH 7.5) and 50 mM K_2_SO_4_), eluted at 0.5 ml/min, monitored at 280 nm. The peak fractions containing α_3_β_3_γ complex were pooled, adjusted to 65% saturated ammonium sulfate, and stored in suspension at 4° C. Approximately 15 mg of α_3_β_3_γ complex was obtained from a 1-L culture. Purified α_3_β_3_γ complex did not contain bound nucleotides (<0.06 mol/mol) as measured by HPLC [43]. The α_3_β_3_γ complex was collected by centrifugation and dissolved in 50 mM Tris-H_2_SO_4_ (pH 7.5) and 50 mM K_2_SO_4_.

The WT ε subunit of BF_1_ was purified as described previously [42], and the mutant ε^133C^ subunit was purified as follows. Approximately 3 g of BL21(DE3)/pET21-BF1-ε(133C) cultivated as described previously [42], was suspended to ~0.2 g of wet cells/ml in buffer D (50 mM Tris-HCl (pH 8.0), 1 mM EDTA, 1 mM DTT, and protease inhibitor cocktail (Roche Diagnostics)) and then disrupted twice using a French Press. The cell lysate was centrifuged at 3,000 × *g* for 10 min at 4° C to remove cell debris, and the supernatant was centrifuged at 180,000 × *g* for 1 h at 4° C. The rest of the procedures was carried out at 25° C. The supernatant was applied to a DEAE Toyopearl column (40 ml, Tosoh) equilibrated with buffer D. The flow-through fractions containing the ε^133C^ subunit were collected and solid ammonium sulfate was added to 65% saturation. The precipitate was stored at 4° C. The protein was collected by centrifugation at 6,000 × *g* for 15 min at 4° C and dissolved in 30 mL of buffer D containing 10% saturated ammonium sulfate and applied to a butyl Toyopearl column (20 mL; Tosoh) equilibrated and washed with the same buffer. The ε^133C^ subunit was eluted with buffer D at a flow rate at ~3 ml/min and fractions containing the ε^133C^ subunit were pooled, and solid ammonium sulfate was added to 65% saturation and stored at 4° C. Approximately 40 mg of ε^133C^ was obtained from a 1-L culture. The ε^133C^ subunit was collected for analysis by centrifugation and dissolved in 50 mM Tris-H_2_SO_4_ (pH 7.5) and 50 mM K_2_SO_4_.

### ATPase assay

ATPase activity was measured spectrophotometrically with an ATP-regenerating system coupled to NADH oxidation at 25 °C [44]. The assay mixture (1.5 ml) consisted of 50 mM Tris-H_2_SO_4_ (pH 7.5), 50 mM K_2_SO_4_, 2 mM phosphoenolpyruvate, 2 mM MgSO_4_, 0.2 mM NADH, 50 µg/ml pyruvate kinase, 50 µg/ml lactate dehydrogenase, and the indicated concentration of ATP-Mg (equimolar mixture of ATP and MgSO_4_) was transferred to a glass cuvette. Absorbance at 340 nm was measured using a V-550 spectrophotometer (JASCO) at 0.5 or 1-s intervals. The α_3_β_3_γ complex with or without ε subunit was added 2 min after starting the measurements. The mixture was stirred with a magnetic stirrer for 5 s before and after the addition of α_3_β_3_γ complex. The rate of ATP hydrolysis was determined from the rate of NADH oxidation. The final concentration of α_3_β_3_γ complex was 30 nM when measuring ATPase activity in the absence of lauryldimethylamine oxide (LDAO). Typically, 15 µl of 3 µM α_3_β_3_γ complex solution was added to 1.5 ml of the assay mixture. When ATPase activity was measured in the presence of LDAO, the final concentration of α_3_β_3_γ complex was reduced to 3 nM. In that case, 0.1 mg/ml bovine serum albumin (BSA) was included in stock α_3_β_3_γ complex solution (450 nM) to avoid the adsorption of α_3_β_3_γ complex on the plastic tube. Ten microliters of α_3_β_3_γ complex solution (450 nM) was added to 1.5 ml of the assay mixture without LDAO. Then, LDAO (final concentration 0.1%) was added and the solution was stirred continuously. When the ATPase activity of α_3_β_3_γε complexes was measured, the ε subunit was included in the α_3_β_3_γ complex stock solution at a 1:10 (3 µM α_3_β_3_γ complex and 30 µM ε in the absence of LDAO) to 1:100 (450 nM α_3_β_3_γ complex and 45 µM ε in the presence of LDAO) molar ratio. Reaction rates were determined at 2–7 s (initial) and 12–13 min (steady-state) after adding BF_1_. The reaction rate in the presence of LDAO was determined 100–150 s after the addition of LDAO.

### Preincubation with MgADP

The effect of preincubation with MgADP was determined as follows: BF_1_ (10 µM α_3_β_3_γ complex ± 100 µM ε) in 50 mM Tris-H_2_SO_4_ (pH 7.5), 50 mM K_2_SO_4_, and 4 mM MgSO_4_ was mixed with an equal volume of 2× MgADP (equimolar mixture of ADP and MgSO_4_) and incubated for more than 10 min at 25° C (Mg^2+^ concentration was in 2 mM excess ADP). Nine microliters of the mixture was added to 1.5 ml of ATPase assay mixture containing 2 mM MgATP (30 nM α_3_β_3_γ complex ± 300 nM ε). The initial rate (2–4 s after the addition of BF_1_) was determined in this experiment.

### Crosslinking γ and ε subunits

Crosslinking of the γ subunit to the extended conformation of the ε subunit in α_3_β_3_γ^S3C^ε^133C^ was performed as follows. Ammonium sulfate suspensions of α_3_β_3_γ^S3C^ complex and ε^133C^ were centrifuged individually at 20,000 × *g* for 15 min at 4° C. Each precipitate was dissolved in 50 mM Tris-H_2_SO_4_ (pH 7.5) and 50 mM K_2_SO_4_, and 10 mM DTT was added and incubated for 10 min at 25° C. The α_3_β_3_γ^S3C^ and ε^133C^ were mixed at a 1: 10 molar ratio and incubated for 15 min at 25° C. Excess ε^133C^ was removed by ultrafiltration with a centrifugal concentrator (Amicon Ultra, 100-kDa cutoff). The sample was concentrated to approximately 10-fold and ultrafiltration was repeated 3 times after the addition of the same buffer to the original volume. The sample (1 mg/ml) was incubated with or without 4 mM MgATP for 10 min at 25° C; the solution was divided into two tubes, and an equal volume of 100 µM CuCl_2_ or the buffer was added. After 1-h incubation at 25° C, 10 mM EDTA was added to terminate the reaction. After 10 min, 0.1% SDS and 15 mM *N*-ethyl maleimide were added. The samples were analyzed using non-reducing SDS-PAGE (12% acrylamide). Part of the sample without ATP and with CuCl_2_ was saved after the addition of 10 mM EDTA for the ATPase assay. A combination of WT α_3_β_3_γ complex and ε^133C^ served as the control. During the ATPase assays, 50 mM DTT was added to reduce crosslinking between the γ and ε subunits at the time indicated in the figure.

### Other methods

Protein concentrations were determined by the method of Bradford [45] using BSA as a standard. DNA sequences for all of the recombinant proteins were confirmed using an ABI 3130xl Genetic Analyzer (Applied Biosystems). Non-reducing PAGE was performed according the method of Laemmli [46]. Chemicals were of the highest grade available. Kinetic data analyses were performed using Spectra Manager (JASCO) and OriginPro 8.5 and 9.0 (OriginLab), and the kinetic parameters are expressed with standard errors.

## Results

### ATPase activity of BF_1_ and the effect of ε subunit

**Figure 1:**
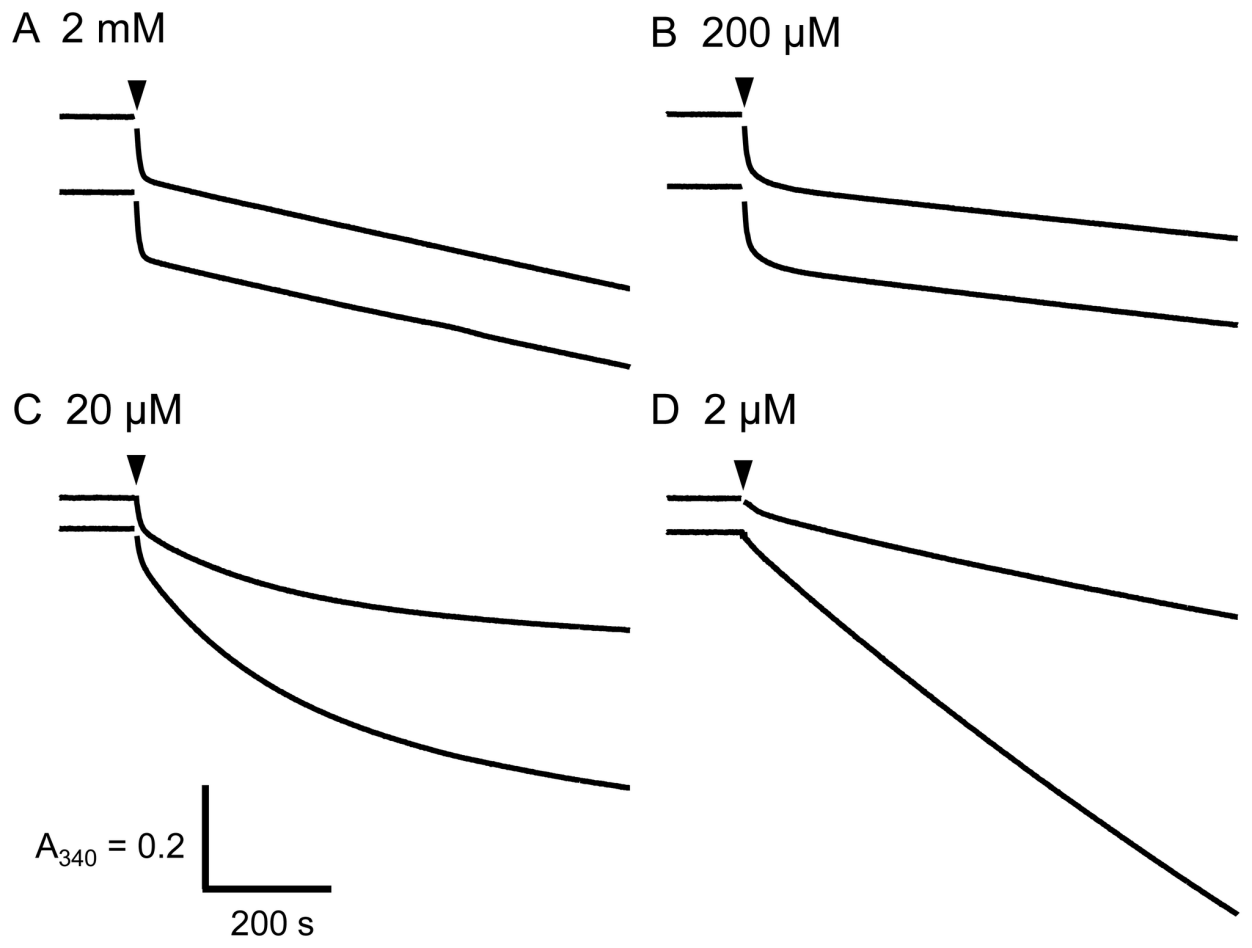
Time-course of ATP hydrolysis by BF_1_ with or without the ε subunit. In each panel, the upper and lower traces represent α_3_β_3_γ and α_3_β_3_γε, respectively. The final concentration of α_3_β_3_γ or α_3_β_3_γε complex of BF_1_ was 30 nM. The ATP concentrations are indicated in the figure. The α_3_β_3_γ or α_3_β_3_γε complex of BF_1_ was added at the time indicated by the arrowheads. The vertical and horizontal bars denote 0.2 absorbance units and 200 s, respectively.

**Figure 2:**
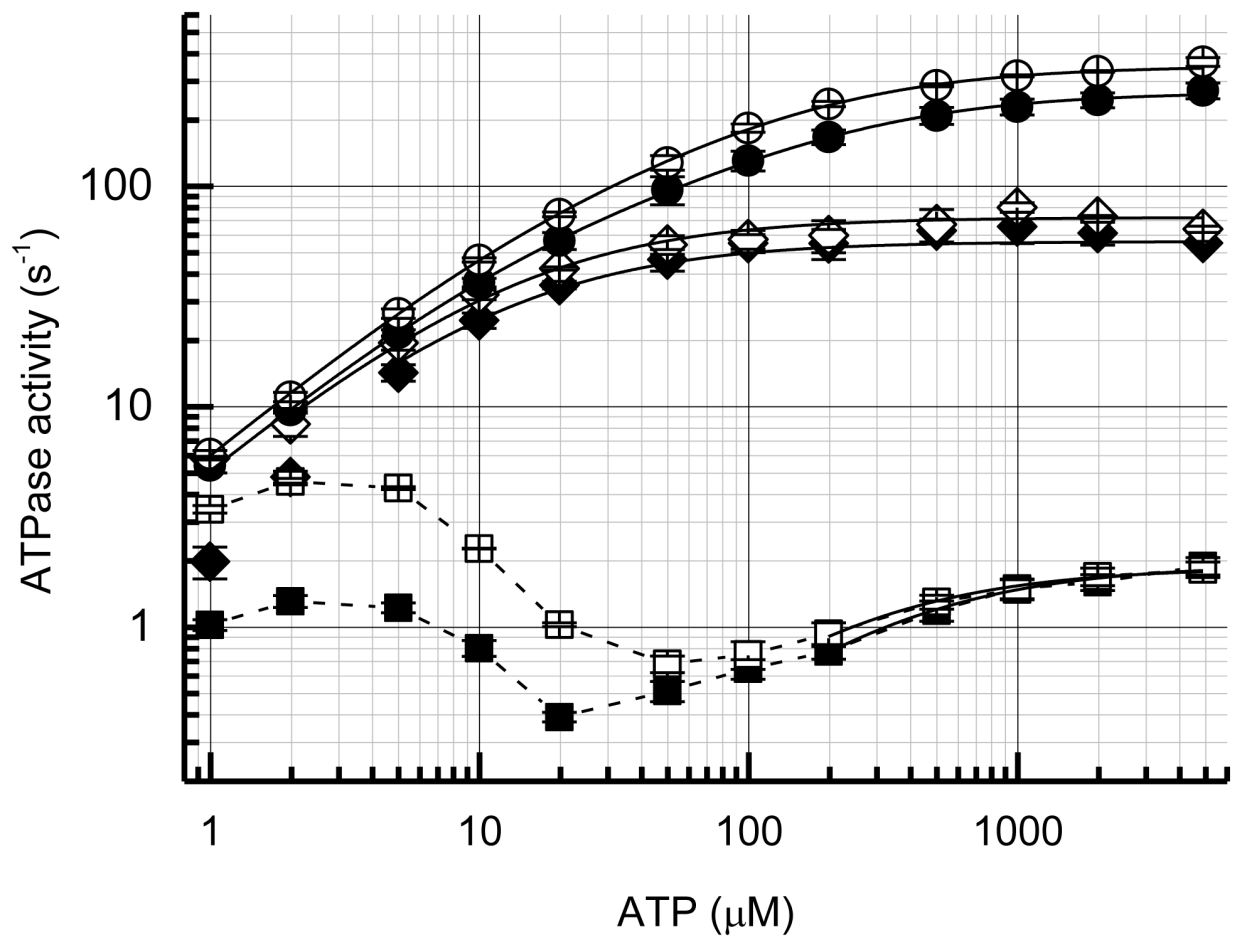
Dependence of BF_1_ ATPase activity on ATP concentration. The ATPase activities of initial (*closed diamonds*; α_3_β_3_γ, and *open diamonds*; α_3_β_3_γε), steady-state (*closed squares*; α_3_β_3_γ, and *open squares*; α_3_β_3_γε) and in the presence of LDAO (*closed circles*; α_3_β_3_γ, and *open circles*; ATPase activities of α_3_β_3_γε) at each ATP concentration was calculated from the velocities at 2-7 s, 12–13 min after the start of the reaction, and 100–150 s after the addition of LDAO, respectively. Error bars represent standard errors. The solid lines were fitted to a single (initial and steady-state) or sum of two (in the presence of LDAO) Michaelis–Menten equation(s). Only data from 200 µM and the above concentrations of ATP were used to fit the steady-state rates of α_3_β_3_γ and α_3_β_3_γε. Data from 1 µM (and 2µM, in the case of α_3_β_3_γ) were not used to fit the initial rate. The *K*_M_ and the associated *k*_cat_ values are 12.7 ± 0.9 µM, 56.2 ± 0.9 s^-1^ (α_3_β_3_γ, initial); 13.8 ± 0.9 µM, 72.3 ± 1.3 s^-1^ (α_3_β_3_γε, initial); 296 ± 25 µM, 1.92 ± 0.06 s^-1^ (α_3_β_3_γ, steady-state); 209 ± 18 µM, 1.87 ± 0.04 s^-1^ (α_3_β_3_γε, steady-state); 16.0 ± 1.9 µM, 68.8 ± 10.9 s^-1^ and 184 ± 32 µM, 199 ± 10 s^-1^ (α_3_β_3_γ, +LDAO); and 18.7 ± 3.4 µM, 80.1 ± 19.2 s^-1^ and 138 ± 18 µM, 272 ± 18 s^-1^ (α_3_β_3_γε, +LDAO).

Typical time courses of ATP hydrolysis by α_3_β_3_γ and α_3_β_3_γε complexes of BF_1_ are shown in Figure 1. At ATP concentrations ≥ 20 µM, very large initial inactivation was observed, irrespective of the presence of the ε subunit. At ATP concentrations > 50 µM, the inactivation was rapid enough to achieve constant, steady-state ATPase activity within the measurement (13 min), and there were no significant differences between α_3_β_3_γ and α_3_β_3_γε at ATP concentrations > 200 µM (Figure 1A, B). At lower ATP concentrations, the rate of inactivation slowed and did not reach the steady state (Figure 1C). Under these conditions, the ATPase activity of α_3_β_3_γε (lower traces in Figure 1) was higher than that of α_3_β_3_γ. Inactivation was diminished at lower ATP concentrations (Figure 1D). Reaction rates determined at 2-7 s and 12–13 min as a function of ATP concentration are shown in Figure 2. The steady-state ATPase activity of BF_1_ exhibited a decrease between 10 and 100 µM ATP possibly due in part to slow inactivation that did not reach the steady-state at low ATP concentrations. The value of *k*_cat_ (1.83 s^-1^ for α_3_β_3_γ and 1.80 s^-1^ for α_3_β_3_γε) for steady-state ATPase activity is very low compared with the F_1_-ATPases from other sources, for example, TF_1_ ~60 s^-1^ and EF_1_ ~75 s^-1^ at 25° C) [39,47]. The ATPase activity increased more than 100-fold by LDAO, which is known to relieve MgADP inhibition (Figure 2). Because the initial rate of ATP hydrolysis reached only about 80 s^-1^ (Figure 2), and 200 mM Pi, which is known to reduce MgADP inhibition [48], activated BF_1_ to only ~10-fold (data not shown), the effect of LDAO may not be entirely related to MgADP inhibition. Nevertheless, these findings indicate that the ATPase activity of α_3_β_3_γ and α_3_β_3_γε complexes of BF_1_ was highly suppressed by MgADP inhibition. Judging from the activation ratio by LDAO, the degree of MgADP inhibition is low at low ATP concentrations. This could account for the triphasic dependence on ATP concentration dependence of ATPase activity in the absence of LDAO in part. In the presence of LDAO, the concentration-dependence on ATP of ATPase activity followed simple sum of two Michaelis–Menten equations (Figure 2).

The ε subunit affected the ATPase activity of BF_1_ only at low concentrations of ATP (Figures 1 and 2). Surprisingly, no inhibitory effect of the ε subunit was observed, and activation by the ε subunit occurred at ATP concentrations <50 µM (Figure 1C, D, lower trace). The dissociation of ε subunit from α_3_β_3_γε complex may not account for the equvialent activities of α_3_β_3_γ and α_3_β_3_γε at high ATP concentrations, because the α_3_β_3_γε complex could be isolated by gel-filtration HPLC (Superdex 200 10/300GL) even in the presence of ATP and/or LDAO (data not shown). Further, the addition of up to 30 µM ε subunit to the ATPase assay mixture did not significantly affect steady-state ATPase activity at 2 mM ATP (data not shown). In the presence of LDAO, the ATPase activities of α_3_β_3_γ and α_3_β_3_γε were essentially the same at all ATP concentrations, although the *k*_cat_ value of α_3_β_3_γε (352 s^-1^) was slightly higher than that of α_3_β_3_γε (268 s^-1^) (Figure 2). The initial rates of ATP hydrolysis were also not significantly different (Figure 2). Therefore, the inhibition by the ε subunit of BF_1_ might be very weak, if any.

### Preincubation with MgADP

**Figure 3:**
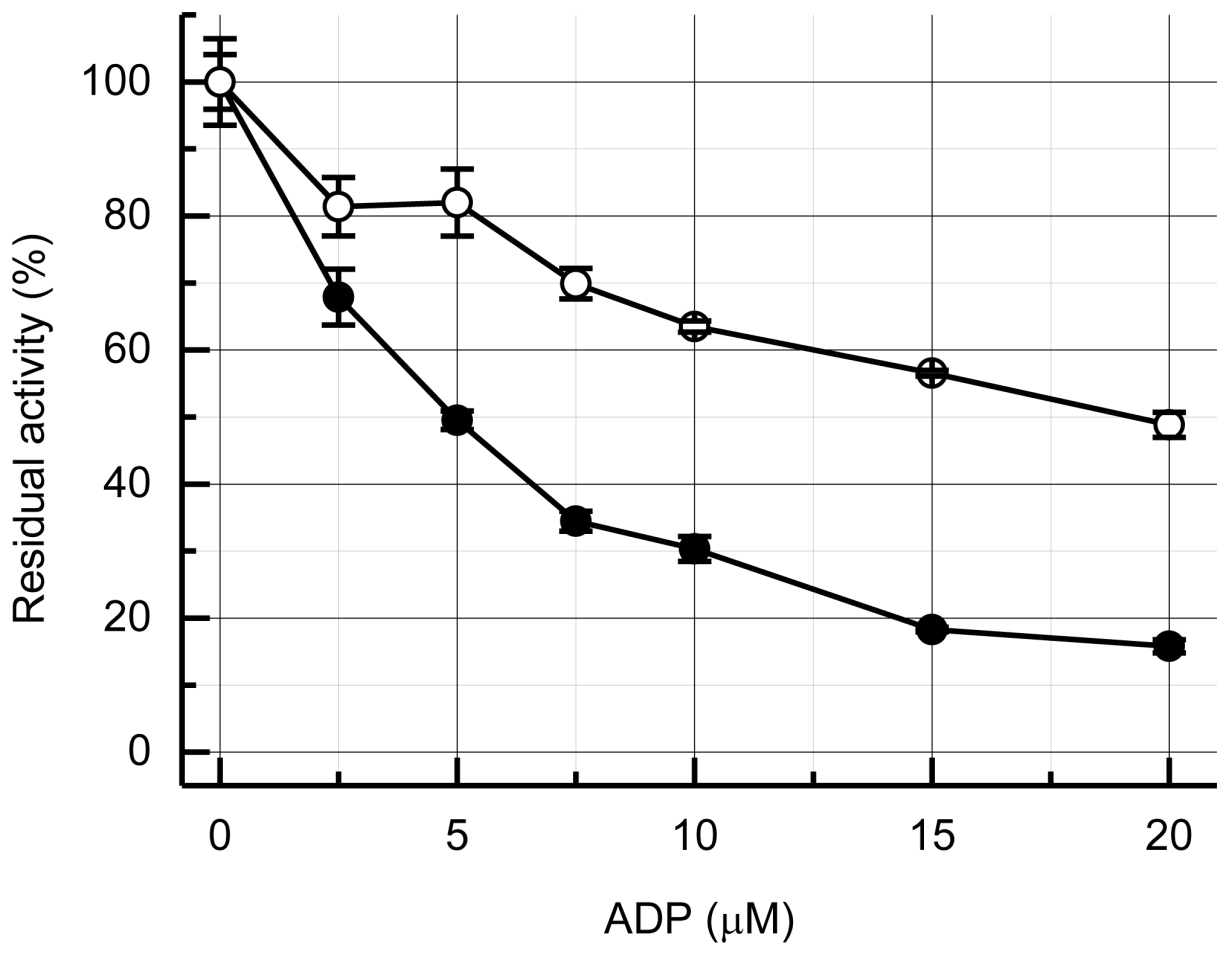
Effect of preincubation with MgADP. The α_3_β_3_γ or α_3_β_3_γε (5 µM) was incubated with the indicated concentrations of MgADP for more than 10 min at 25° C. Residual ATPase activity was measured in the presence of 2 mM ATP. The initial rate (2–4 s after the start of the reaction) was measured, and the values relative to the control without incubation with MgADP (82.9 ± 5.4 s^-1^ and 88.6 ± 3.6 s^-1^ for α_3_β_3_γ and α_3_β_3_γε, respectively) are plotted. Closed and open circles represent α_3_β_3_γ and α_3_β_3_γε, respectively. Error bars represent standard errors.

When α_3_β_3_γ was preincubated with MgADP, the initial ATPase activity was significantly inhibited (Figure 3, closed circles), as reported previously for other F_1_-ATPases [26,48]. Incubation of 5 µM α_3_β_3_γ with 1:1 and 1:2 MgADP resulted in about 50% and 70% inhibition, respectively. In the presence of the ε subunit (open circles), the ATPase activity of α_3_β_3_γε was inhibited only marginally compared with that of α_3_β_3_γ; incubation with 1:1 and 1:2 MgADP resulted in about 20% and 35% inhibition, respectively. Reduced inhibition by the preincubating α_3_β_3_γε with MgADP might be due to the suppression of MgADP binding by the ε subunit in its extended conformation.

### Catalytic properties of mutant BF_1_ with its ε subunit fixed in the extended conformation

**Figure 4:**
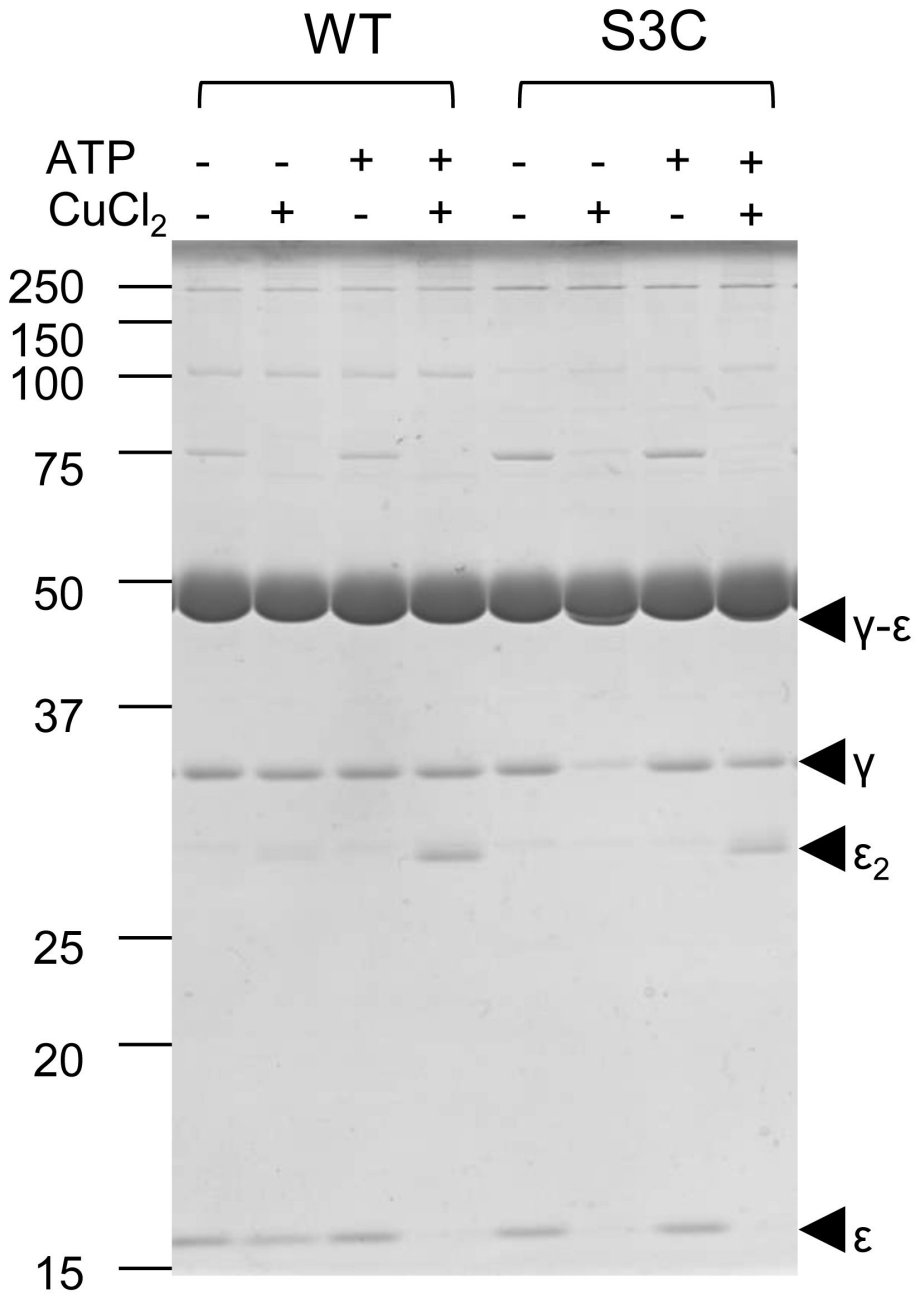
Non-reducing SDS-PAGE analysis of mutant α_3_β_3_γ^S3C^ε^133C^. The α_3_β_3__γ_^WT^_ε_^133C^ (WT) or α_3_β_3_γ^S3C^ε^133C^ (S3C) were incubated for 1 h at 25°C with combinations of 2 mM ATP and 50 μM CuCl2 as indicated at the top of the figure. After the incubation, the samples were subjected to non-reducing SDS-PAGE (12% acrylamide). Bands derived from γ and ε subunits are marked by arrowheads.

The activation by the ε subunit was investigated in more detail by examining a mutant α_3_β_3_γε complex of BF_1_, in which the N-terminus of the γ subunit and C-terminus of the ε subunit can be crosslinked via engineering in Cys residues to fix the ε subunit in the extended conformation. Thus, a mutant equivalent to that of TF_1_ [13] was prepared. The endogenous Cys residues in the α subunit did not react with the introduced Cys residues in the γ or ε subunits. To determine whether the apparent absence of ε inhibition in BF_1_ resulted from the inability of the ε subunit to assume an extended conformation, the presence of γ-ε crosslink formation was determined in the presence or absence of ATP. The γ and ε bands disappeared and a band corresponding to the γ-ε crosslink product appeared only in the absence of ATP (Figures 4, and S2). The distance between the Cα of the residues corresponding to the introduced Cys residues in a recently reported EF_1_ structure is 12.9 Å [16]. Although this is a little bit long to form a disulfide bridge, the formation of a disulfide bridge within the mutant α_3_β_3_γ^S3C^ε^133C^ complex of BF_1_ indicates that the crosslinked structure may reflect the physiological conformation within the range of thermal fluctuation. In the presence of ATP, a dimer of the ε subunit was formed, indicating that ε changed its conformation from the extended to the intermediate or folded-state in which the C-terminal Cys was accessible on the surface of the molecule. We conclude from these results that the absence of ε inhibition was not caused by the absence of its extended conformation. However, because there were no significant differences in the initial activities of WT α_3_β_3_γ and α_3_β_3_γε complexes, the extended conformation of the ε subunit may readily change upon addition of ATP.

**Figure 5:**
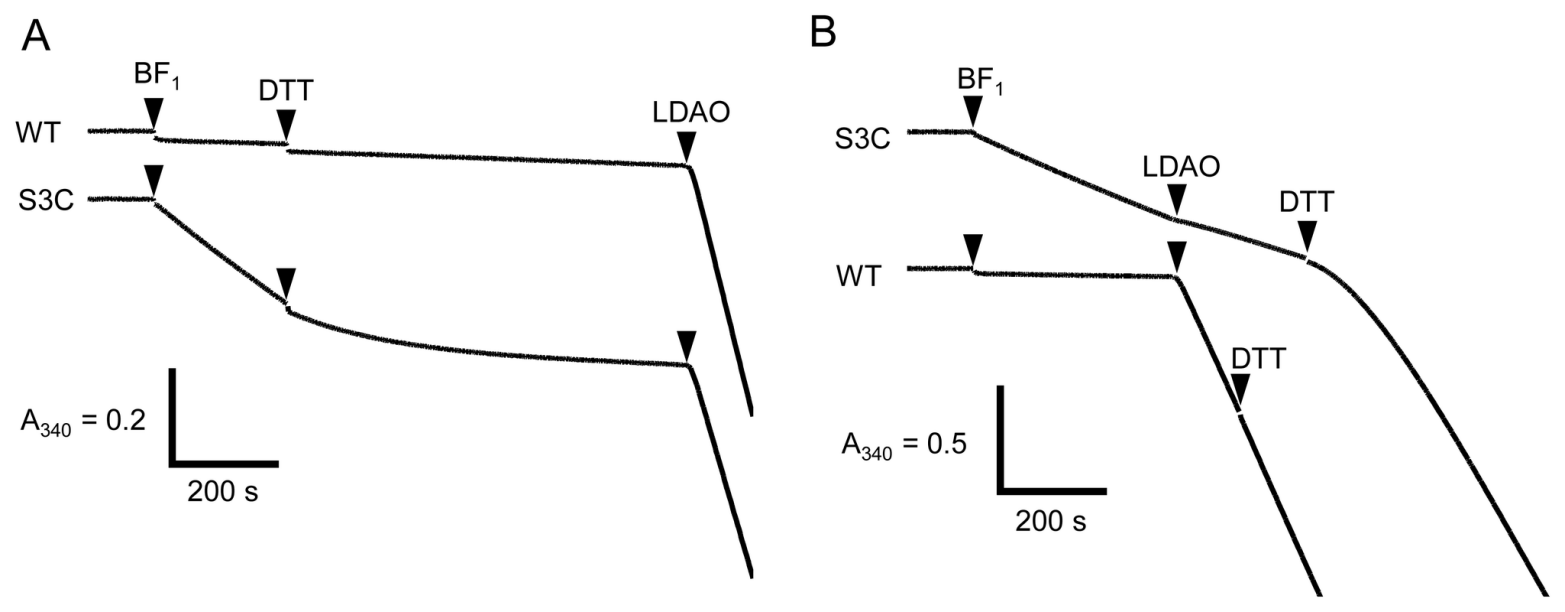
Kinetics of ATP hydrolysis by mutant α_3_β_3_γ^S3C^ε^133C^. ATPase activities of α_3_β_3_γ^S3C^ε^133C^ (S3C), and α_3_β_3_γ^WT^ε^133C^ (WT) at 2 mM ATP were determined. The α_3_β_3_γε complex of was added to 3 nM at the times indicated by the first arrowheads. (*A*) DTT (50 mM) and LDAO (0.1%) were added at the times indicated by the second and third arrowheads, respectively. (*B*) The order of addition of DTT and LDAO was reversed.

We next determined the ATPase activity of the crosslinked mutant α_3_β_3_γ^S3C^ε^133C^. In the absence of LDAO, the activity of the mutant was significantly higher than that of WT, even at 2 mM ATP (Figure 5A). When crosslinking was reduced by the addition of 50 mM DTT, the activity gradually decreased to the same level as WT. The addition of LDAO after this reduction resulted in activation to the WT level. When LDAO was added before reduction, activation of ATPase activity was undetectable (Figure 5B). The subsequent addition of DTT resulted in full activation.

## Discussion

### ATPase activity of BF_1_ is strongly suppressed by MgADP inhibition

BF_1_ showed high initial ATPase activity, rapid inactivation, and very low steady-state ATPase activity. LDAO dramatically activated the steady-state ATPase activity of BF_1_ (Figure 2). The initial ATPase activity was >20-fold higher than the steady-state ATPase activity, 200 mM Pi also activated steady-state ATPase activity by ~10-fold (data not shown), and preincubation with MgADP greatly suppressed the initial ATPase activity (Figure 3). These suggest that the inactivation might be due to strong MgADP inhibition, which could mean that the *B. subtilis* ATP synthase functions as an ATP synthase that does not hydrolyze ATP, because MgADP inhibition does not inhibit ATP synthesis activity [49].

### No inhibition by the ε subunit

The ε subunit did not significantly inhibit the ATPase activity of BF_1_ but activated at low concentrations of ATP presumably due to the suppression of MgADP inhibition. Further, the ε subunit, fixed in the extended conformation, did not inhibit the mutant enzyme. The ε subunit only inhibited the activity of the extended-state fixed mutant α_3_β_3_γ^S3C^ε^133C^ complex of BF_1_ in the presence of LDAO (Figure 5B, S3C after addition of LDAO). In this case, DTT activated the enzyme, indicating that the activity before the addition of DTT was actually suppressed by the extended-state ε subunit. We conclude, therefore, that due to the strong MgADP inhibition, ε inhibition is not evident, because the relief from MgADP inhibition by the ε subunit is more prominent.

### Counteraction of MgADP inhibition and ε subunit

As discussed above, the ε subunit suppressed MgADP inhibition. In the absence of LDAO, the mutant α_3_β_3_γ^S3C^ε^133C^ complex of BF_1_ with an extended-state fixed ε subunit showed considerably higher ATPase activity than the WT, even at 2 mM ATP. LDAO did not activate the ATPase activity (Figure 5B). Thus, even before the addition of LDAO, the extended-state fixed α_3_β_3_γ^S3C^ε^133C^ complex of BF_1_ might be less inhibited by MgADP inhibition. This conclusion is further strengthened by the results when the WT BF_1_ was preincubated with MgADP (Figure 3). In the absence of the ε subunit, preincubation with MgADP suppressed the ATPase activity of α_3_β_3_γ proportionally at a α_3_β_3_γ: MgADP ratio of 1:2, suggesting that binding of MgADP is strong and binding of one or two MgADP is enough to induce MgADP inhibition of α_3_β_3_γ complex. In contrast, greater than 60% of the activity was retained in the presence of 1:2 MgADP and the ε subunit, indicating that binding of MgADP to α_3_β_3_γε was highly suppressed by the ε subunit. This agrees well with our previous observation that the ε subunit of TF_1_ significantly suppresses the binding of MgADP [33]. LDAO did not activate the extended-fixed α_3_β_3_γ^S3C^ε^133C^ complex of BF_1_ (Figure 5B), indicating that the extended-state ε subunit reduced MgADP inhibition. Considering all of these results, we conclude that ε inhibition is not due to the stabilization of MgADP inhibition [28,29,31], but due to an essentially different and counteracting mechanism. We believe, therefore, that these properties must be common among various F_1_-ATPases, despite the differences in the mechanisms of ε inhibition. It should be noted, however, the ε subunit did not protect mutant α_3_β_3_γ^S3C^ε^133C^ complex from MgADP inhibition by the preincubation with ADP (Figure S1). These apparent contradictory results may be due to the different catalytic site affinity for nucleotides between WT and the mutant (γ^S3C^) α_3_β_3_γ complexes, and/or different mode of the action of MgADP during preincubation and ATPase turnover *etc*. Further experiments, for example, measurement of nucleotide binding to the catalytic sites with WT and mutant α_3_β_3_γ with and without ε subunit will give us a clue to resolve the differences between WT and the mutant in the MgADP preincubation experiment.

### Significance of regulation by the ε subunit and MgADP inhibition in vivo

The results presented here indicate that the ATPase activity of BF_1_ is very low under normal conditions due to strong MgADP inhibition. Because *B. subtilis* lives in an aerobic environment and its ATP synthase is primarily used to synthesize ATP but not to hydrolyze ATP, as is the case for bacteria such as *E. coli* that can grow anaerobically. The ε subunit may not act as an inhibitor of the ATPase activity of *B. subtilis* ATP synthase. In contrast, its ability to attenuate MgADP inhibition may be its primary role in the regulatory system. Experiments using *B. subtilis* with mutant FoF_1_ to address these questions are underway in our laboratory. Elucidation of the balance and the interplay of these two regulatory systems in different bacteria may be required to understand the regulation of bacterial ATP synthases.

## Supporting Information

Figure S1 **Effect of preincubation with MgADP on α_3_β_3_γ^S3C^ε^133C^.**
The α_3_β_3_γ^S3C^, DTT-treated α_3_β_3_γ^S3C^ε^133C^ and CuCl_2_-treated α_3_β_3_γ^S3C^ε^133C^ (5 µM) were subjected to the same experiment as shown in Figure 3. Closed circles, open circles and open squares represent α_3_β_3_γ^S3C^, DTT-treated α_3_β_3_γ^S3C^ε^133C^ and CuCl_2_-treated α_3_β_3_γ^S3C^ε^133C^, respectively. Error bars represent standard errors.
(TIF)

Figure S2 **Enlargement of the part of Figure 4.**
The part of Figure 4 is enlarged to visualize γ-ε crosslinked band clearer. Only the region around α, β and γ-ε from α_3_β_3_γ^S3C^ε^133C^ (S3C) treated with or without CuCl_2_ in the absence of ATP is shown.
(TIF)
