# Supplementary figures and images for: ε Subunit of Bacillus subtilis F1-ATPase Relieves MgADP Inhibition
Source: PLoS One. 2013 Aug 13;8(8):e73888. doi: 10.1371/journal.pone.0073888 (PMC3742539; doi:10.1371/journal.pone.0073888)

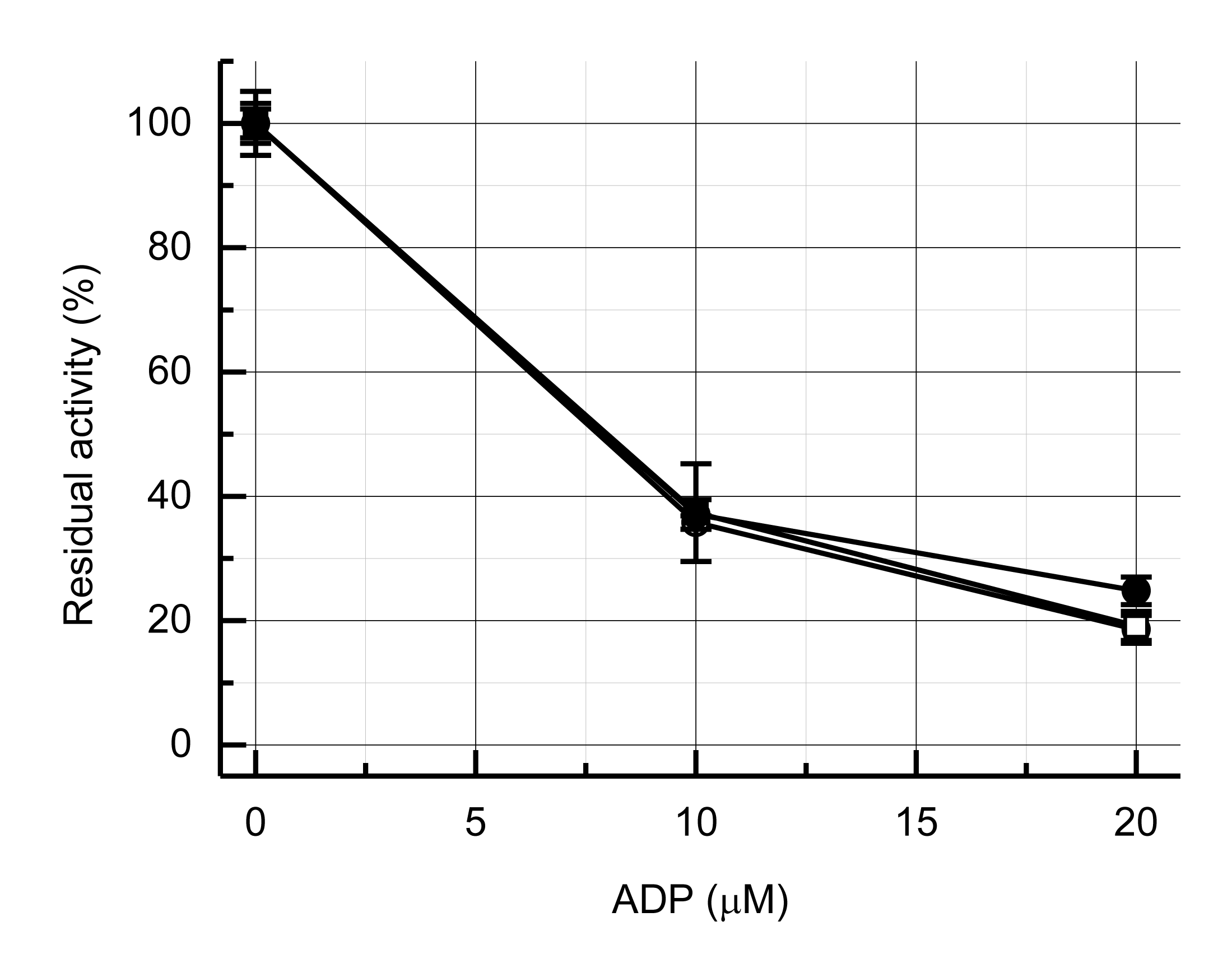

Supplement: Figure S1 — Effect of preincubation with MgADP on α3β3γS3Cε133C. The α3β3γS3C, DTT-treated α3β3γS3Cε133C and CuCl2-treated α3β3γS3Cε133C (5 µM) were subjected to the same experiment as shown in Figure 3. Closed circles, open circles and open squares represent α3β3γS3C, DTT-treated α3β3γS3Cε133C and CuCl2-treated α3β3γS3Cε133C, respectively. Error bars represent standard errors. (TIF) [file pone.0073888.s001.tif]

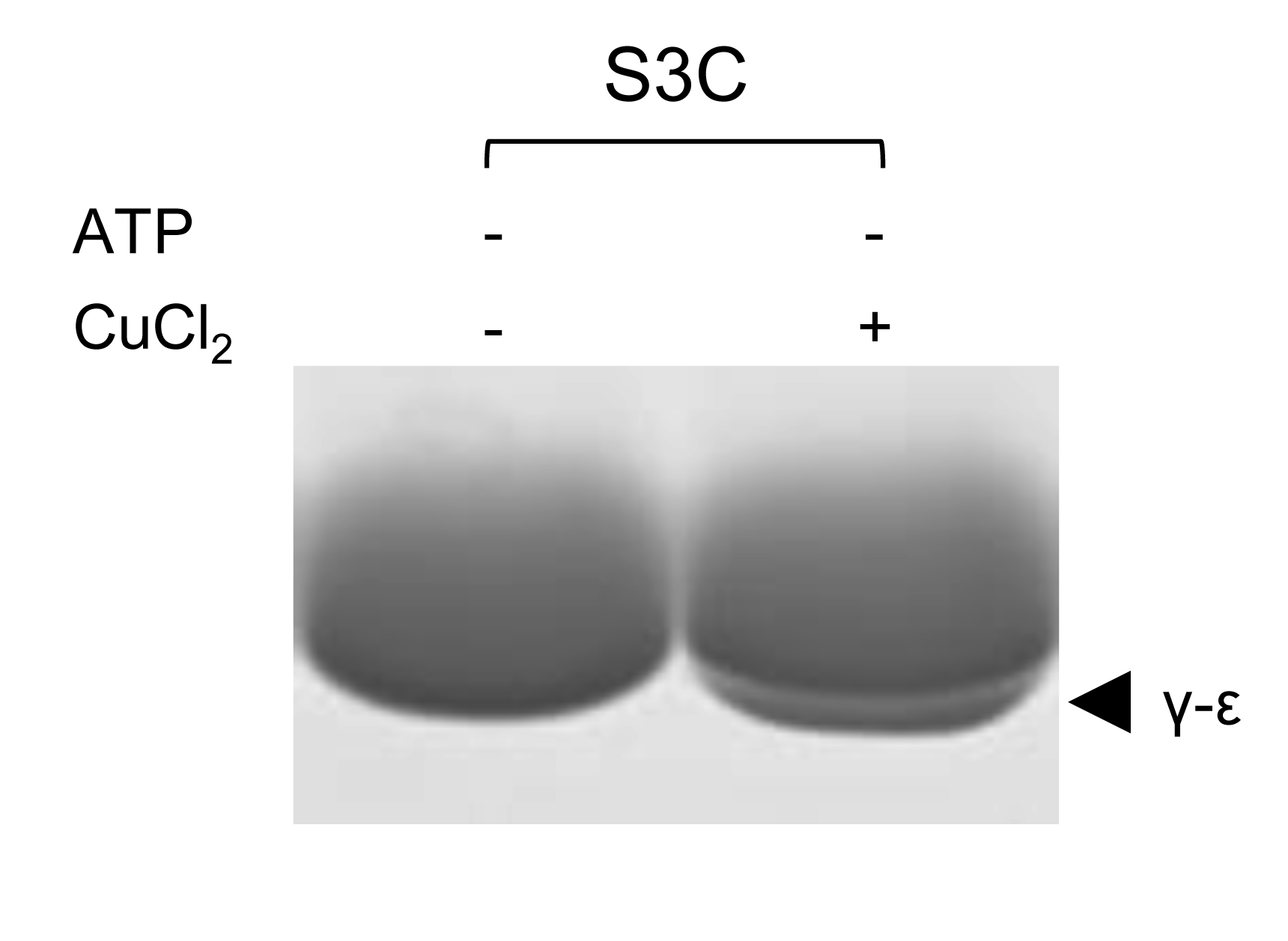

Supplement: Figure S2 — Enlargement of the part of Figure 4. The part of Figure 4 is enlarged to visualize γ-ε crosslinked band clearer. Only the region around α, β and γ-ε from α3β3γS3Cε133C (S3C) treated with or without CuCl2 in the absence of ATP is shown. (TIF) [file pone.0073888.s002.tif]
